# Data-driven vermiculite distribution modelling for UAV-based precision pest management

**DOI:** 10.3389/frobt.2022.854381

**Published:** 2022-08-10

**Authors:** Na Ma, Anil Mantri, Graham Bough, Ayush Patnaik, Siddhesh Yadav, Christian Nansen, Zhaodan Kong

**Affiliations:** ^1^ Department of Mechanical and Aerospace Engineering, University of California, Davis, Davis, CA, United States; ^2^ Department of Entomology and Nematology, University of California, Davis, Davis, CA, United States

**Keywords:** precision agriculture, precision pest management, unmanned aerial vehicles, machine learning, data-driven model

## Abstract

In recent decades, unmanned aerial vehicles (UAVs) have gained considerable popularity in the agricultural sector, in which UAV-based actuation is used to spray pesticides and release biological control agents. A key challenge in such UAV-based actuation is to account for wind speed and UAV flight parameters to maximize precision-delivery of pesticides and biological control agents. This paper describes a data-driven framework to predict density distribution patterns of vermiculite dispensed from a hovering UAV as a function of UAV’s movement state, wind condition, and dispenser setting. The model, derived by our proposed learning algorithm, is able to accurately predict the vermiculite distribution pattern evaluated in terms of both training and test data. Our framework and algorithm can be easily translated to other precision pest management problems with different UAVs and dispensers and for difference pesticides and crops. Moreover, our model, due to its simple analytical form, can be incorporated into the design of a controller that can optimize autonomous UAV delivery of desired amount of predatory mites to multiple target locations.

## 1 Introduction

**FIGURE 1:**
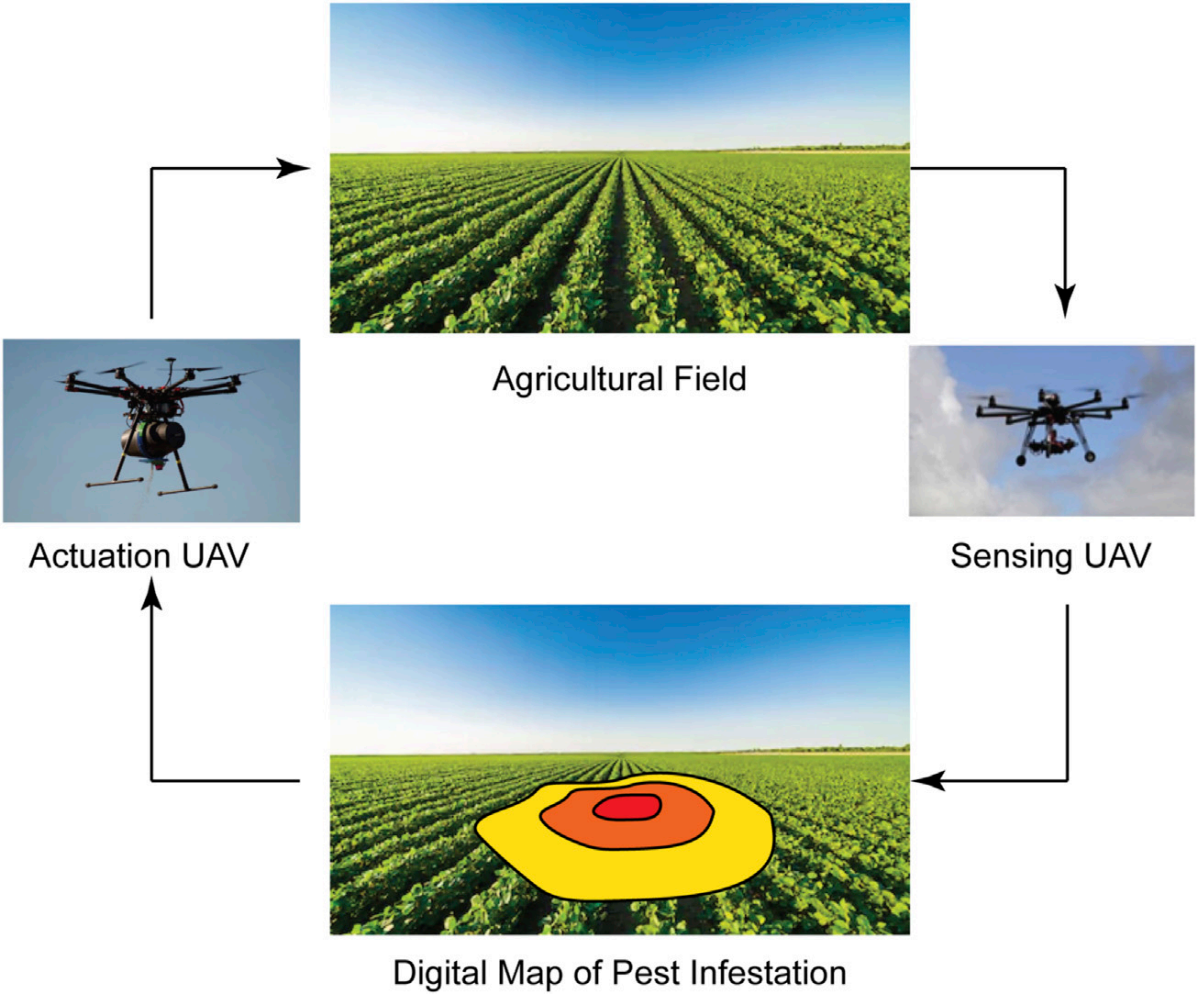
Our envisioned closed-loop UAV-based precision pest management system. This system features two types of UAVs (Teske et al., 2019; Iost Filho et al., 2020): (1) sensing UAVs to scan the field and identify infested plants (Nansen et al., 2019, 2021) and (2) actuation UAVs to precisely and autonomously deliver persimilis to the infested plants.

Examples of actuation with unmanned aerial vehicles (UAVs) include collection of water and air samples (West and Kimber, 2015; Koparan et al., 2018), and in agriculture, UAV-based actuation includes pesticide spray applications (Shim et al., 2009; Giles and Billing, 2015; Xue et al., 2016; Xiongkui et al., 2017; Yallappa et al., 2017; Yun et al., 2017; Ukaegbu et al., 2021) and distribution of biological control agents (Spoorthi et al., 2017; Iost Filho et al., 2020; Zhan et al., 2021). There are two basic reasons why UAV-based actuation solutions are being developed and commercialized for agricultural applications: 1) perceived reductions in operating costs, as UAV-based actuation may replace labor intensive procedures, and 2) as part of promotion of precision agriculture, higher likelihood of precision-delivery of pesticides and/or biological control agents to within-field hotspots with emerging pest outbreaks (see Figure 1). Increased precision-delivery of pesticides and/or biological control agents may lead to reductions in operating costs and less risk of agricultural practices having adverse environmental and health effects on surrounding non-agricultural environments and urban areas. Several important and multi-faceted factors appear to support the argument that UAV-based actuation will play a major role in many aspects of 21st century agriculture: 1) UAV technology is continuing to improve in terms of flight performance, hardware durability, batteries and flight duration, and maximum payload, 2) there is an increase in ways to obtain necessary training and certification, 3) legislation and permits to fly UAVs are becoming clearer and easier to comply with, and 4) costs of UAV systems are either stagnant or declining.

Despite the abovementioned justifications supporting widespread acknowledgement of the promises and potential of UAV-based actuation, it is equally important to highlight current challenges and shortcomings. In both pesticide applications and distributions of biological control agents, one of the main challenges is directly linked to complex interactions between 1) UAV flight (speed, direction and altitude), 2) wind (speed and direction relative to UAV flight path), and 3) actuated objects (size, shape, and density of spray droplets and/or biological control agents). In a series of studies, Qin et al. (Qin et al., 2014, 2016) collected data on the overall distribution uniformity of liquid droplets within crop canopies and then used wind speed, wind direction, UAV altitude, and UAV speed as explanatory variables of droplet distribution uniformity. Their model was able to successfully characterize cumulative distribution of liquid pesticides, but their model did not characterize the spatial droplet distribution. Teske et al. (Teske et al., 2019) developed and tested a model to characterize the one-dimensional (perpendicular to the flight path) distribution of vermiculite as a function of wind speed and direction, and the UAVs altitude and forward speed. Using these explanatory variables, model validation produced an average generalisation error of 12.8%, RMSE. Optimization of UAV actuation may rely on existing modeling of these complex interactions, as similar challenges are being faced when UAVs are used to deliver and drop supplies during rescue and disaster missions (Restas, 2015; Gupta et al., 2020) and distribution of fire retardants to wildfires (Ausonio et al., 2021).

In this study, we investigated the hypothesis that the two-dimensional distribution of a low-density material, vermiculite^1^, from a hovering UAV can be accurately modeled based on the following explanatory variables: UAV altitude, wind speed, wind direction, and the dispenser settings (aperture size and flow rate). Vermiculite was chosen as model substance as it is a lightweight (low-density) mineral and therefore highly susceptible to flight parameters and environmental conditions. Additionally, vermiculite is often used as carrier in UAV-based distributions of biological control agents. Although the focus in this study is on vermiculite, we believe the model framework presented has broad relevance to other UAV-based actuation applications involving low-density materials.

## 2 Materials and methods

In this section, we will describe a series of experiments we have conducted to systematically collect data that can sufficiently characterize the relationship between the vermiculite distribution and a collection of independent factors/variables, e.g., UAV altitude, wind speed and direction, and vermiculite dispenser settings.

### 2.1 Experiment hardware and software

The UAV used in this study was a DJI SPREADING WINGS S1000 + Octocopter. It was retrofitted with a PIXHAWK2 UAV Autopilot Flight Controller with a HERE+ Vehicle GPS rover module and a telemetry module. A FrSky X8R ACCST Telemetry Receiver was also mounted on the UAV so that the UAV can also be manually controlled with a handheld Radio Control (RC) transmitter.

**FIGURE 2:**
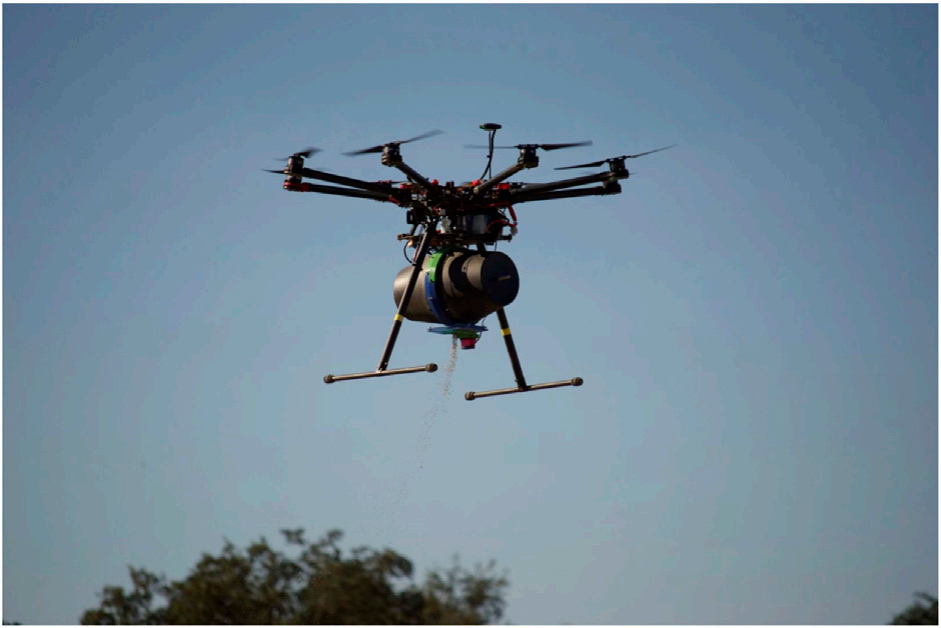
DJI S1000 + octocopter with the vermiculite dispenser “Bugbot” attached.

**FIGURE 3:**
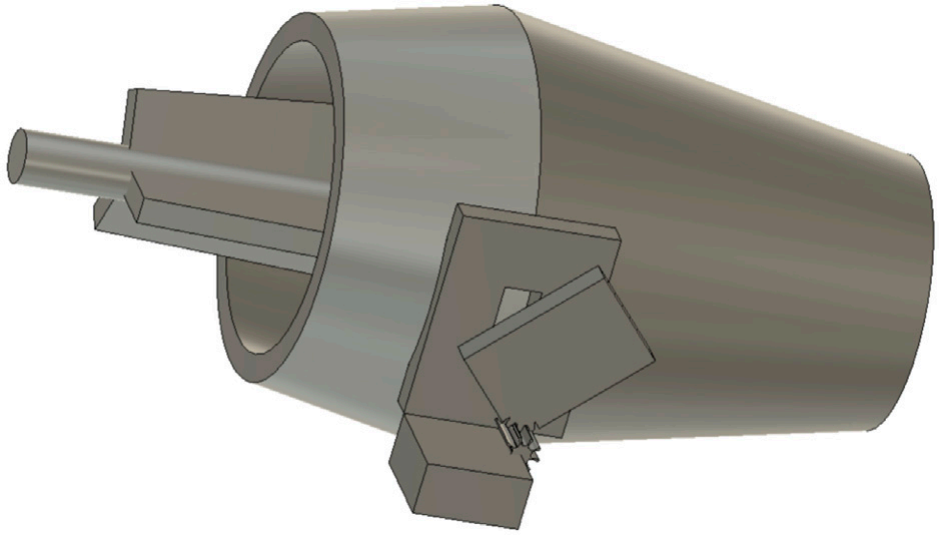
Computer Aided Design (CAD) model of our “Bugbot”.

A custom-designed vermiculite dispensing mechanism called “Bugbot” was attached underneath the UAV (see Figure 2) The “Bugbot”, designed specifically for UAVs to deliver predatory mites (*Phytoseiulus persimilis*) to target locations, consists of three major components: a vermiculite container, an internal mixing mechanism, and a 3D printed dispensing mechanism. It is an updated version of our previous design (Teske et al., 2019) with the following four improvements: 1) The container was changed from a 3.5-liter polyhedron with multiple edges and corners to a 12-liter horizontal rotational symmetric container, 2) The new container is made of a material that is less likely to generate static electricity, making the vermiculite flow smoother. 3) The dispenser was changed from a motor-driven constant-speed paddle wheel to a gear and motor-driven slide plate, which can not only be opened at any angle through Wi-Fi control to easily dispense different volumes, but also completely eliminated the problem of the old design where vermiculite would be stuck and stop the paddle wheel during the operation. 4) The mixing mechanism was a first-time design that does not exist in the older version, and it can mix the vermiculite with the predatory mites for a more uniformed distribution from the dispenser. Although the flow rate has only changed from the original 5.67 g/s to 5.95 m/s, which seems to be a small improvement, but our new Bugbot design offers a bigger container volume, a more uniform dispensing flow rate, and the ability to change the dispensing volume. Figure 3 shows the Computer Aided Design (CAD) model of our Bugbot.

Two symmetric cone-shape parts are joined together with 3D printed parts on the circumference to form the Bugbot container body. On top of circumference is a thick acrylic mount base that is used to attach the Bugbot to the UAV. Vermiculite can be refilled through a capped opening on the side of the container. The dispensing mechanism consists of an acrylic plate that can rotate to uncover a rectangular hole on a second acrylic plate, is attached to the bottom of the container. The amount of covered, ranging from fully open to fully closed, is controlled by a servo motor and gears cut on the moving acrylic plate. The motor was controlled by an Arduino Nano AT32 hardwired to a Raspberry Pi, both mounted on the UAV, by commands to rotate the acrylic plate to predefined opening sizes for predefined opening times sent through a local WiFi network. In this way, the vermiculite dispensing volume can be controlled by different combinations of opening areas with opening duration.

Once added to container as a mixture with vermiculite, predatory mites may move and aggregate, which can lead to non-linear correlation between dispensing of vermiculite and predatory mites. Static electricity inside the container may exacerbate this issue. Accordingly, constant mixing of the vermiculite is required. Therefore, a rotational mechanism was designed inside the container with a steel rod passes through the center of the container horizontally, and a continuous 360-degree servo motor of 30 kg rating at the middle of the rod which can rotate the rod at a constant speed. Two sets of steel fins were attached to this continuously rotating rod, one set on each side of the container, to help mix the vermiculite. This mixing mechanism provides two benefits: 1) a more uniform dispensing flow-rate and 2) movement of the vermiculite to ensure the predatory mites are evenly distributed during flight and dispensing.

### 2.2 Field data collections

Field data were from September to October 2021 in Davis, California. Wind speed and direction data were recorded using a YOUNG Model 91,000 ResponseONE Ultrasonic Anemometer, with a sampling frequency of 20 Hz. The anemometer was installed on a telescopic antenna push up mast and placed around the same height as the UAV’s flight altitudes in the experiments. For accuracy purpose, the anemometer was placed at a short distance away from the UAV’s flight path and its altitude remaining unchanged during field data collection events. Weather data included wind speed (*w*) data in m/s and wind direction (*α*) data in deg. They can be easily converted to wind velocity components *w*_*E*_ and *w*_*N*_ in East and North directions: *w*_*E*_ = −*wsin*(*α*) and *w*_*N*_ = −*wcos*(*α*). The experimental design was not aligned with North-South direction: the positive *y* direction of the boards, as mentioned in Section 2.2, is *λ* = 157 deg to the North. To make collected data more intuitive, wind data were converted to two components along the *x* and *y* axes: *w*_*x*_ = *w*_*E*_ *cos*(*λ*) − *w*_*N*_ *sin*(*λ*) and *w*_*y*_ = *w*_*E*_ *sin*(*λ*) + *w*_*N*_ *cos*(*λ*).

**FIGURE 4:**
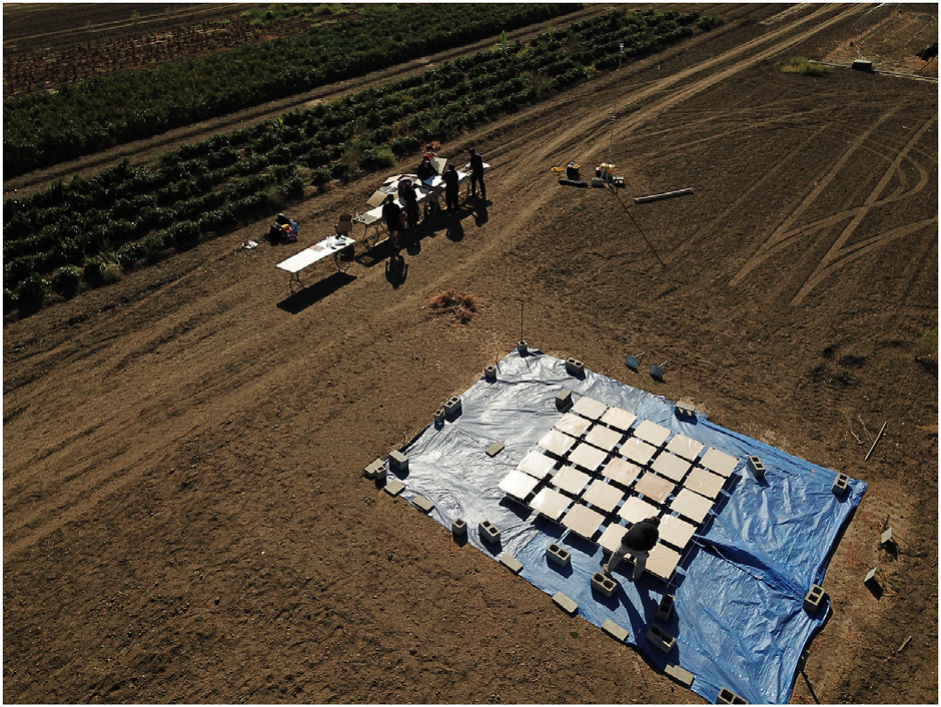
Experiment site and equipment setup.

**FIGURE 5:**
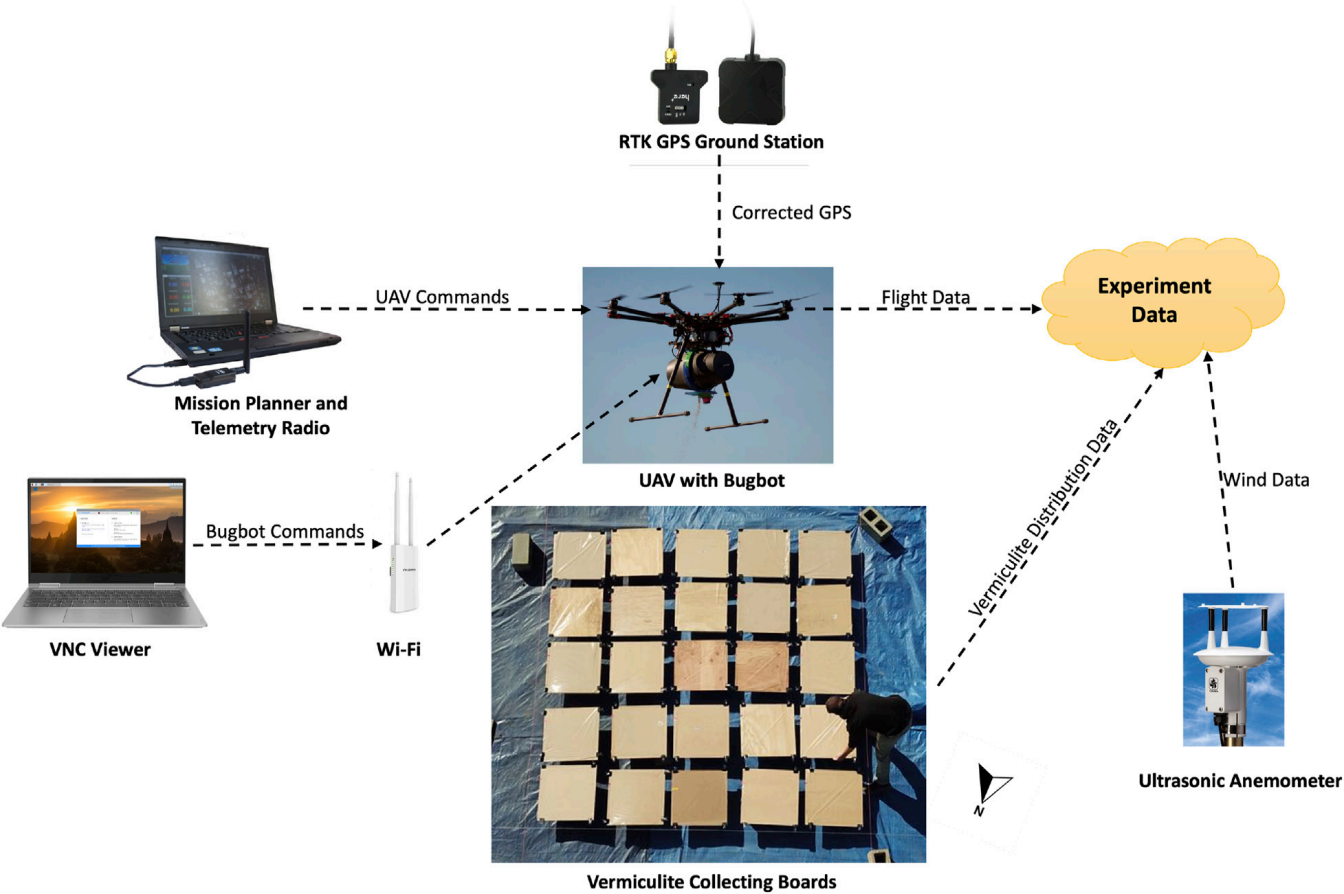
A block diagram illustrating our data collection system.

During experimental dispensing events, the UAV was kept hovering. The hovering location was automatically controlled by waypoints set up in a software called Mission Planner on Windows. Since the changing wind condition affected the stability of the UAV when it was hovering, to ensure the accuracy of the waypoint when the UAV was dispensing the vermiculite, the UAV’s PID controller’s parameters were tuned with the presence of the wind before all experiments. Furthermore, a real time kinematics (RTK) capable HERE+ Ground Station GPS was set up at a fixed location on a tripod with sufficient sky coverage at the experiment field to obtain good satellite signals and generate accurate position estimates in centimeters in real time. In order to collect ground distribution data of vermiculite dispensed from the hovering UAV, outdoor experiments were carried out from Figure 4 shows the experimental field site and equipment setup. During each dispensing event, the UAV was controlled by the Mission Planner to hover at a waypoint above with the same yaw angle in all experimental flights. The RTK GPS was calibrated before each flight event to ensure the accuracy of the UAV’s hovering waypoints. A Wi-Fi router was set up at the field for the computer software VNC Viewer to communicate with the Bugbot and send dispensing commands. Figure 5 shows our data collection system.

Plywood boards of 0.6-m square, each with 0.15-m gap from each other for easy placement and labeling, were placed in a 5 by 5 grid on top of 25 cinder blocks on a tarp. Elevation of boards away from the ground was performed to minimize cross-contamination by dirt or grass or accumulated vermiculite on the ground. In order to make the dispensed light-weight vermiculite stay at place on the boards and prevent them from being blown away by the wind or by the vortex from the UAV propellers, each board was covered with carpet protection tape, with the adhesive side facing up, and secured with binder clips. After each vermiculite dispensing event, a 0.6-m square newsprint paper was placed over each of the 25 adhesive tapes to cover and protect vermiculite collected.

In each dispensing event, the UAV hovered at the center of the five-by-five grid of boards with the same, and dispensed vermiculite for 3 s. The independent variables of our experiments were: 1) the UAV’s altitudes *h*, 2) the dispenser’s opening areas *S*, and 3) the wind speed *w* and directions *α*. We tested two different UAV altitudes: 3.5 and 4 m and two different dispenser opening area: 100 and 75%. The altitudes were chosen to be these values so that they were neither too high that the wind would blow most of the vermiculite off the boards area, nor too close to the boards that the collected data would be affected by the vortex created by the UAV’s propellers. The dispenser opening areas were chosen based on measurements of vermiculite masses dispensed with different openings (for 3 s). The dispensed masses of 100 and 75% opening areas were 17.85 and 7.56 g, respectively, which were suitable values for our analysis purpose. Each combination of UAV altitude and dispenser opening area were tested multiple times on several days with different wind conditions. A total of 22 vermiculite dispensing trials were performed. A video of the experiment setup and data collection process is on YouTube at: https://youtu.be/st_apuEBtJg.

### 2.3 Image analysis and data calibration

**FIGURE 6:**
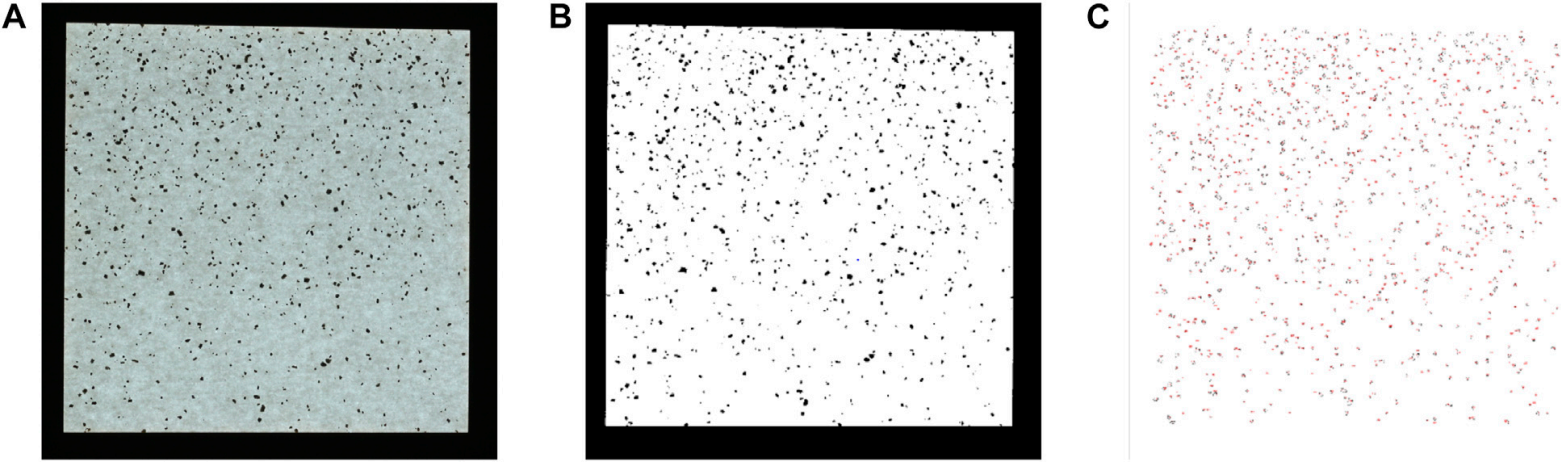
Image analysis: **(A)** is an original vermiculite sample picture taken by a camera. **(B)** is the corresponding image edited by ImageJ for analysis. **(C)** shows the vermiculite particles detected by ImageJ.

Field data of vermiculite distribution on adhesive paper were analyzed based on image analysis. Moreover, each of the 0.6-m square data papers was divided into four square quadrants and the inner 0.2-m square in each quadrant was imaged on a lightboard. Each quadrant was labeled with its row, column, trial number, and orientation information in the field, which can map to its location on the grid of boards. The lightboard can illuminate the empty space around the vermiculite, making the vermiculite appear in dark contrast to the background (see Figure 6A). The images were then analyzed using a software called ImageJ, where the image analysis process was automated to perform the following analysis on each image: 1) convert it to a binary image (see Figure 6B), 2) crop, and 3) count the dark particle areas (see Figure 6C). For the particle analysis in ImageJ, a circularity value of 0.30–1.00 was chosen to eliminate artifacts, such as wrinkles, and contamination, such as insects or grass on a paper. The size of particles counted was set to 0–10,000 square pixels, which represented the size range of the medium grade vermiculite used.

In order to relate vermiculite mass in each quadrant to the pixel area detected by ImageJ, a calibration experiment was carried out. Calibration samples were created using the same carpet protector tape used in the experiments and covered with the same newsprint paper on the adhesive sides. Three duplicate calibration quadrants with vermiculite in the inner 0.2-m square area were made for each of the 10 different vermiculite masses: 0, 0.010, 0.020, 0.040, 0.080, 0.160, 0.320, 0.640, 1.280, and 2.560 g. These values were chosen according to the distribution of pixel areas analyzed on all the outdoor experiment data, with the most of papers having smaller pixel areas and fewer with larger pixel areas. The smallest measurement taken was with no vermiculite to account for noisy pixel areas from the non-uniformity of the paper’s material. The largest mass was chosen so that it had a larger total pixel area than that of the maximum value among all outdoor experiment quadrants. A balance with 0.001 g precision was used to measure the masses of vermiculite used in the calibration experiment.

**FIGURE 7:**
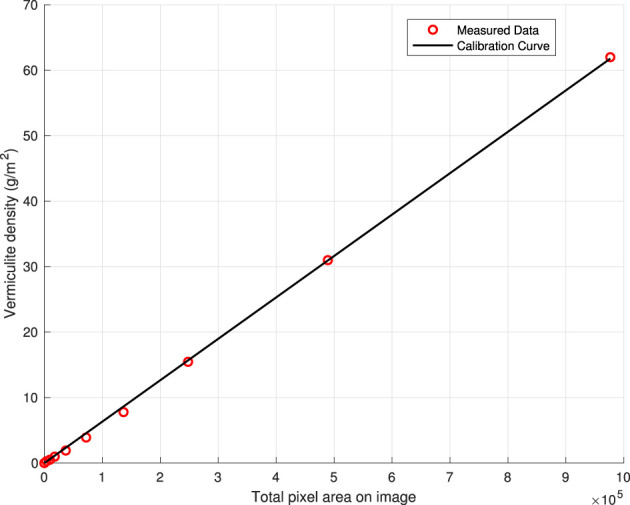
Vermiculite density calibration curve: vermiculite density versus pixel area detected by ImageJ.

A linear calibration curve (slope *s*_*c*_ was 6.3249 × 10^–5^ g/m^2^ with an R-squared value of 0.9996) showed that image analusis could be used to accurately estimate vermiculite density in g/m^2^ with each image’s pixel area (see Figure 7).

## 3 Data-driven vermiculite distribution modelling approach and results

Based on wind speed and direction, UAV altitude, and dispenser opening area as explanatory variables, a 2D distribution (i.e., the spatial density function) model of vermiculite dispersed by a hovering UAV was developed. Firstly, we fit the experimental field data (22 flight missions and data from 25 boards from each event) to a Gaussian function; secondly, standard machine learning was deployed to optimize model parameters.

### 3.1 Gaussian distribution fitting

**FIGURE 8:**
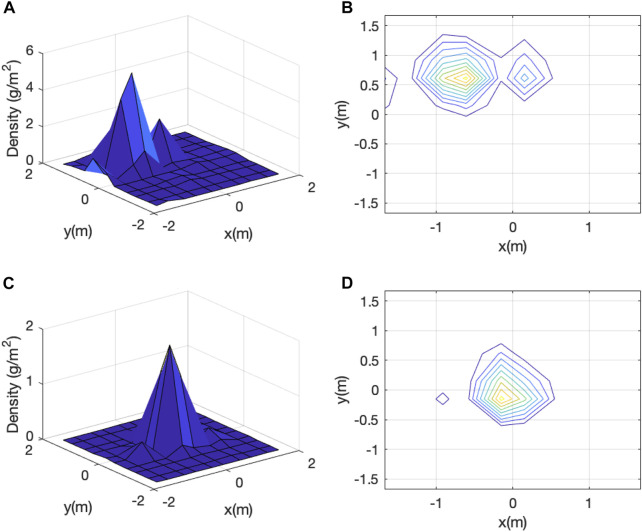
Raw vermiculite density data of two dispensing trials: **(A)** and **(C)** show the 3D plots and **(B)** and **(D)** show the corresponding contour plots.

Figure 8 shows two representative vermiculite distribution plots. One, shown in Figures 8C,D, exhibits only one peak while the other, shown in Figures 8A,B, exhibits two peaks with one more pronounced than the other. Out of all 22 trials, 18 have only one peak, 4 have two peaks, and none has more than two peaks. Moreover, the magnitudes of the second peaks, if they exist, are always less than 30% of those corresponding first peaks. Finally, presence of multiple peaks is likely caused by wind characteristics, mainly changes in wind direction and speed during a trial, as vermiculite is falling from the hovering UAV. Therefore, for the four trials with two peaks, we only retain and study their dominant peaks. We will call the data set with the second peaks removed as “post-processed”.

All post-processed 22 sets of vermiculite density field data possessed characteristics of a bi-variate Gaussian distribution, which can be represented by the following exponential function:

$$d\left(x,y\right)=\frac{s_{c}A}{2\pi \sigma_{x}\sigma_{y}\sqrt{1-\rho^{2}}}\exp\left(-\frac{1}{2\left(1-\rho^{2}\right)}\left[{\left(\frac{x-\mu_{x}}{\sigma_{x}}\right)}^{2}-2\rho \left(\frac{x-\mu_{x}}{\sigma_{x}}\right)\left(\frac{y-\mu_{y}}{\sigma_{y}}\right)+{\left(\frac{y-\mu_{y}}{\sigma_{y}}\right)}^{2}\right]\right).$$ (1)

Here *d* is the density of vermiculite on the ground in g/m^2^. *x* and *y* (each being a function of altitude *h* (m)) are the relative *x* and *y* coordinates of boards in 5 by 5 grids. Positive *y* direction is *λ* = 157 deg to the North direction. *μ*_*x*_ and *μ*_*y*_ are the mean values of *x* and *y*, respectively. *σ*_*x*_ and *σ*_*y*_ are the standard derivations of *x* and *y*, respectively. *ρ* is the correlation between *x* and *y*. *A* denotes peak value in pixel area. Finally, *s*_*c*_ is the slope of the calibration curve mentioned in Section 2.3.

The natural logarithms on both sides of Eq. 1 generates a linear equation of ln *d* (*x*, *y*) with variables *x*, *y*, *xy*, *x* ^2^, and *y* ^2^ (Guo, 2011):

$$\ln d\left(x,y\right)=a_{0}+a_{1}x+a_{2}y+a_{3}xy+a_{4}x^{2}+a_{5}y^{2}.$$ (2)

Accordingly, Gaussian distribution function parameters *A*, *μ*_*x*_, *μ*_*y*_, *σ*_*x*_, *σ*_*y*_, and *ρ* in Eq. 1 have the following relationship with the coefficients *a*_*i*_ (*i* = 0, 1, 2, 3, 4, 5) of the polynomial function in Eq. 2:

$$a_{0}=\ln\left(\frac{As_{c}}{2\pi \sigma_{x}\sigma_{y}\sqrt{1-\rho^{2}}}\right)-\frac{1}{2\left(1-\rho^{2}\right)}\left(\frac{\mu_{x}^{2}}{\sigma_{x}^{2}}+\frac{\mu_{y}^{2}}{\sigma_{y}^{2}}-2\rho \frac{\mu_{x}\mu_{y}}{\sigma_{x}\sigma_{y}}\right)$$ (3)

$$a_{1}=-\frac{1}{2\left(1-\rho^{2}\right)}\left(-\frac{2\mu_{x}}{\sigma_{x}^{2}}+2\rho \frac{\mu_{y}}{\sigma_{x}\sigma_{y}}\right)$$ (4)

$$a_{2}=-\frac{1}{2\left(1-\rho^{2}\right)}\left(-\frac{2\mu_{y}}{\sigma_{y}^{2}}+2\rho \frac{\mu_{x}}{\sigma_{x}\sigma_{y}}\right)$$ (5)

$$a_{3}=\frac{\rho }{\left(1-\rho^{2}\right)\sigma_{x}\sigma_{y}}$$ (6)

$$a_{4}=-\frac{1}{2\left(1-\rho^{2}\right)\sigma_{x}^{2}}$$ (7)

$$a_{5}=-\frac{1}{2\left(1-\rho^{2}\right)\sigma_{y}^{2}}$$ (8)

**FIGURE 9:**
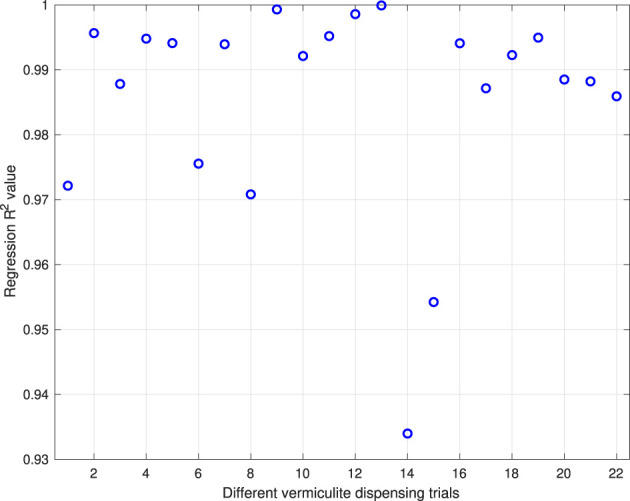
R^2^ values of the linear regression results for all 22 trials.

**FIGURE 10:**
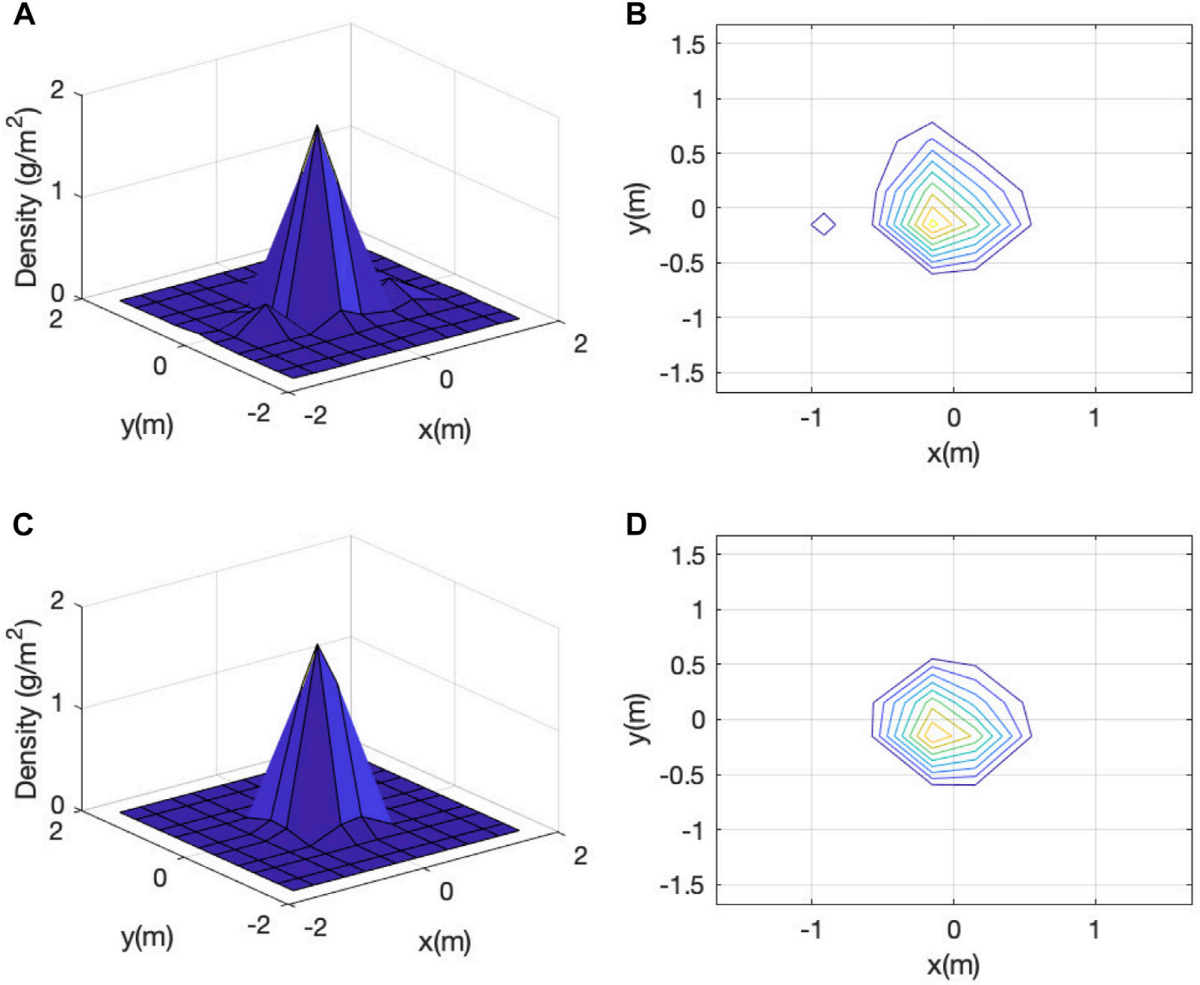
Raw vermiculite density data, **(A)** and **(B)**, versus its fitted Gaussian distribution using linear regression, **(C)** and **(D)**.

One major advantage of Eq. 2, which is linear, over Eq. 1, which is nonlinear, is that the former allows us to apply standard linear least square regression to determine the coefficients $a_{i}^{\prime }s$ . Importantly, *a*_*i*_ values for different flight events vary, due to different combinations of independent variables such as UAV altitude and dispenser settings. To determine individual *a*_*i*_ values for each field data set, locations of each of vermiculite sample quadrant (*x*, *y*) and the natural logarithm of vermiculite density ln *d*(*x*, *y*) on each quadrant are used in the least square regression. Moreover, bi-variate Gaussian distributions of each dispensing trial can be determined by converting ln *d*(*x*, *y*) back to *d*(*x*, *y*). Figure 9 shows the R-squared values of the regression results for all trials. All R^2^ values are above 0.9 which suggests the $a_{i}^{\prime }s$ , determined by the linear regression, are sufficiently precise to represent the raw data’s maximum peak distribution. Figure 10 shows vermiculite distribution of one representative field data set and its corresponding fitted Gaussian distribution.

### 3.2 Distribution coefficients learning

*a*_*i*_ values determine shapes of bi-variate Gaussian distributions for different field data sets. They are affected by the following factors (independent variables): 1) UAV altitude (*h*), 2) wind speeds in *x* and *y* direction (*w*_*x*_ and *w*_*y*_), and 3) Bugbot dispenser opening area (*S*). Given these input vectors $X_{i}≔{\left[c,h,w_{x},w_{y},S\right]}^{\prime }$ and their corresponding output vectors $Y_{i}≔{\left[a_{0},\ldots ,a_{5}\right]}^{\prime }$ (one input-output pair for one trial), a function *f* to minimize a pre-defined cost function, such as a root mean square (RMS) function $C\left(X_{i},Y_{i}\right)≔\sum_{i=1}^{N}{\left(Y_{i}-f\left(X_{i}\right)\right)}^{2}$ was used, in which *N* is the number of trials (22 in our case).

$$f\left(X_{i}\right)=C{\bar{X}}_{i},$$ (9)

where **C** is an unknown matrix that needs to be determined. Prediction accuracy of this matrix may be improved by adding third and higher order terms of *h*, *w*_*x*_, *w*_*y*_, and *S* to the feature vector ${\bar{X}}_{i}$ (Friedman et al., 2001). But this will make the model more expensive to compute online. Moreover, we will demonstrate later that a model with second order features is sufficiently accurate for the precision pest management application.

With *a*_*i*_ values determined in Section 3.1, linear least square regression is used in conjunction with cross-validation for all possible combinations of the 15 terms in ${\bar{X}}_{i}$ (there are $\sum_{n=1}^{15}C_{15}^{n}=32767$ combinations in total). Leave-one-out cross validation was performed on 20 randomly selected trials. The 2 remaining field data sets used for testing (see Section 4.1). For each of the leave-one-out (20-fold in our case) cross-validations, 19 field data sets were used to train the model, and the remaining field data set was used to select the feature combination (out of $C_{15}^{n_{i}}$ ) with the best validation performance. The learned function, i.e., Eq. 9, is as follows:

$$\left\{\begin{array}{c} a_{0}=8.2946w_{y}-0.5412h^{2}+3.5169hw_{x}-16.4139w_{x}S-10.5451w_{y}S\;\\ a_{1}=-2.4065h^{2}-0.2156w_{y}^{2}-28.6857S^{2}+17.0366hS-0.4442w_{x}w_{y}+0.9154w_{x}S-0.5847w_{y}S\;\\ a_{2}=-0.0433w_{y}^{2}-1.1698S^{2}+0.3448hw_{x}-1.9576w_{x}S+0.5137w_{y}S\;\\ a_{3}=-0.0007w_{y}^{2}\;\\ a_{4}=-1.0142-1.8745w_{x}+0.0180w_{x}^{2}+0.5352hw_{x}+0.0370hw_{y}\;\\ a_{5}=0.3376w_{y}-0.0459h^{2}+0.0335w_{x}^{2}+0.0356w_{y}^{2}-0.0691hw_{y}+0.0669w_{x}w_{y}+0.1104w_{x}S\; \end{array}\right.$$ (10)

## 4 Discussion

### 4.1 Model validation

**FIGURE 11:**
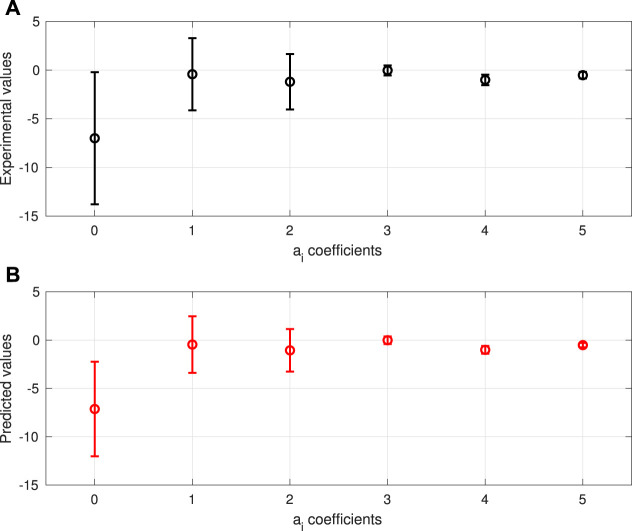
Model performance with respect to the validation data: **(A)**: the mean and standard derivation values of the experimental *a*_*i*_ values (obtained in Section 3.1) and **(B)**: those of the predicted *a*_*i*_ values using Eq. 10.

**FIGURE 12:**
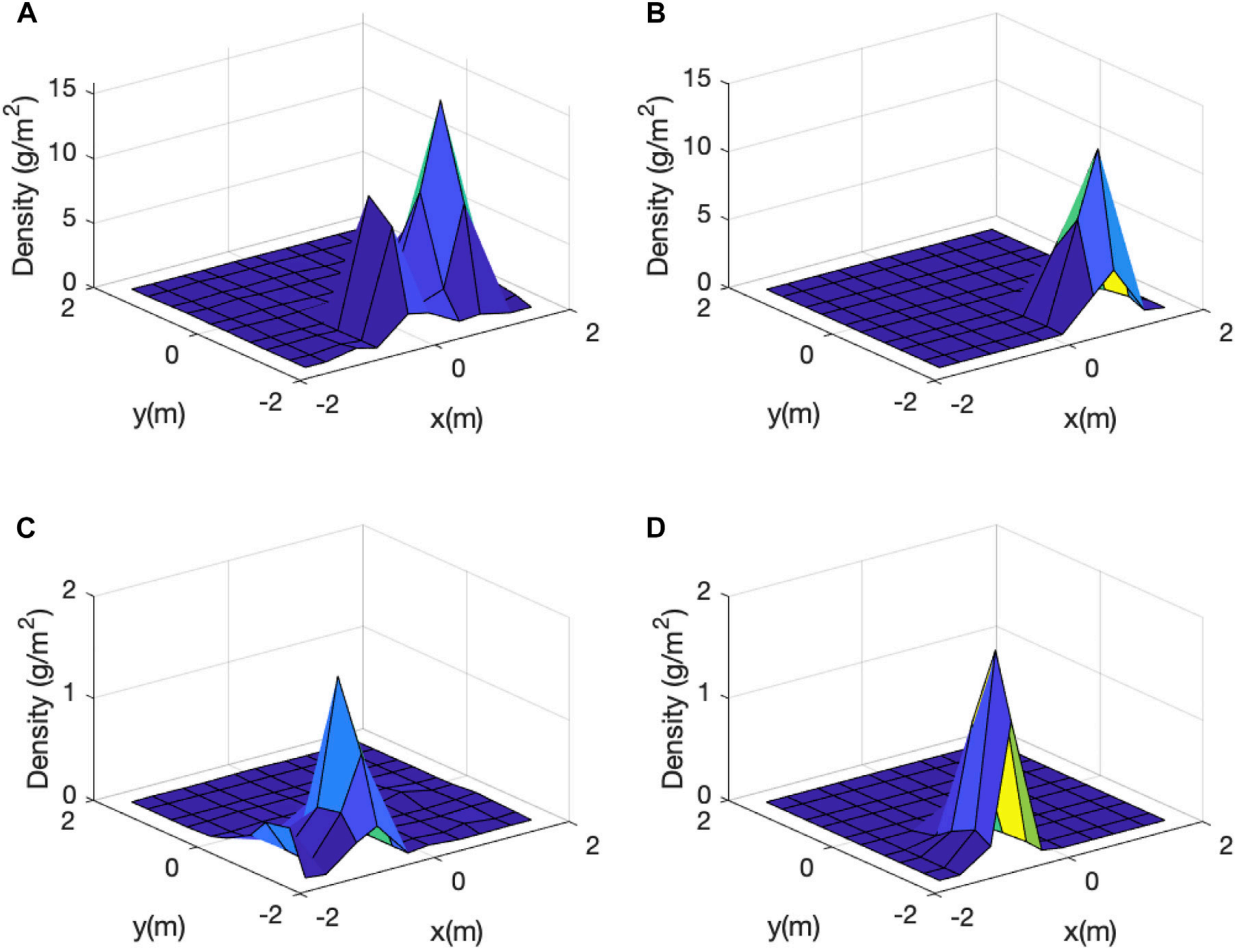
Model performance with respect to the test data: **(A)**: an experimental/raw vermiculite distribution and **(B)**: its predicted distribution based on our learned model (Eqn. 10); **(C)**: another experimental/raw vermiculite distribution and **(D)**: its predicted distribution based on our learned model (Eq. 10).

The learned data-driven model, i.e., Eq. 10, performed well in the validation: the RMS between the $a_{i}^{\prime }s$ obtained from the experiments and those predicted by the model is only 13.91% (see Figure 11). To further evaluate robustness of our learned model, we tested its predictive performance based on test data (the 2 trials that are not included in the model training). Figure 12 show the results for these two field data sets (Figures 12A,B for the first set and Figures 12C,D for the second one), and Table 1 shows the experimental and predicted parameter values for second set (the performance is similar for the first set). Figure 12 shows that location and shape of the predicted distributions aligned well with actual experimental field data. Accordingly, the learned model provided accurate prediction of vermiculite density distribution from a hovering UAV.

**TABLE 1:** Experimental *a*_*i*_ values versus predicted *a*_*i*_ values.

Coefficients	Experimental Values	Predicted Values
*a*_0_	−11.8399	−11.8015
*a*_1_	−7.0722	−7.0755
*a*_2_	−2.5284	−2.5508
*a*_3_	−0.0093	−0.0007
*a*_4_	−1.8349	−1.7993
*a*_5_	−0.4390	−0.2928

### 4.2 Limitations and proposed solutions

During our model training process, only maximum peak of field data set was used. Therefore, in some of the flights (those with two or more peaks removed), the actual dispensed volumes were slightly different from those used in the analysis. Furthermore, we observed that at comparatively higher wind speeds, a small amount of vermiculite was blown to areas off the boards grid (we tried to mitigate the issue by moving grids of boards further downwind). Finally, gaps between boards were left for simplicity to place and label boards. However, this setup made the vermiculite distribution data dis-continuous. A better experiment setup for future studies might be: 1) using continuous boards that can cover a larger area to counter changes in the wind, 2) using shorter dispenser operating period to decrease the effect of the changes in wind conditions on vermiculite distribution, 3) dividing the collected samples into more pieces, rather than the current quadrant, to provide more data points, which may lead to smoother contours and better models (with more data).

This study focused exclusively on effects of wind speed and direction. However, vermiculite dispensing may be, at least partially, influenced by temperature and ambient humidity. Thus in future studies, modeling and parameterisation of these abiotic variables should be added to the experimental design.

This study was based on experimental data collected with a hovering UAV. Future experimental studies are needed, in which flight speed and direction in relation to wind direction are taken into account.

### 4.3 Future controller design and relevant applications

Our learned model, due to its predictive power and simple analytical form, can be easily adapted to other UAV actuation purposes. That is, precision-delivery of materials from UAVs is not only important and restricted to agricultural pest management. Moreover, prototypes for UAV-based package delivery systems are being developed and tested (Romero et al., 2016). In support of rescue emergency operations and disaster management (Restas, 2015), UAVs are used to deliver blood, medications, and other healthcare products to locations inaccessible by roads or water ways. Similarly, innovative skyports can deploy UAVs to deliver to expected locations (Gupta et al., 2020). Another popular use of delivery UAVs is to drop fire retardants or spread extinguishing liquid to fire front, which requires modeling of the wind speed and direction, the payload carried by UAVs, and the time for UAVs to reach the fire front, in order for the materials released to reach the part of the fire front that is best to address the fire extinguishing goal with UAVs (Ausonio et al., 2021). In other situations like nuclear accidents, hazardous material leakages, floods, and earthquakes (Restas, 2015), it is hard and dangerous for humans to get actively involved, UAVs can not only be used to monitor dynamic situations, they can also deliver useful materials to suppress and mitigate risks. To further improve these and similar UAV-based actuation applications, precision delivery is among the most important challenges to be solved, and accurate modeling of wind speed and direction is a cornerstone in such research efforts.

## 5 Conclusion

Dispensing of lightweight materials, such as vermiculite, from a flying or hovering UAV is invariably influenced by UAV flight settings (i.e., speed and altitude) and by wind parameters (i.e. speed and direction in relation to UAV fliht path direction). Without accurate modeling of material distribution as afunction of these explanatory variables, the possibility of precision-delivery is markedly impaired. Based on experimental field data, a data-driven vermiculite distribution model with machine learning techniques was developed and optimized. Results confirmed that 2D vermiculite distribution could accurately predicted from a hovering UAV. The model can be used to optimize the control of UAV pest management, where the vermiculite distribution can be predicted with the proposed model in real time, and the UAV controller can use this information to autonomously deliver desired amount of pesticide to targeted locations.

## Data Availability

The raw data supporting the conclusions of this article will be made available by the authors, without undue reservation.

## Footnotes

1 To keep predatory mites, such as persimilis, alive, a mineral medium, called vermiculite, is used to pack them.
